# Omicron BA.2 (B.1.1.529.2): high potential to becoming the next dominating variant

**DOI:** 10.21203/rs.3.rs-1362445/v1

**Published:** 2022-02-23

**Authors:** Jiahui Chen, Guo-Wei Wei

**Affiliations:** 1 Department of Mathematics, Michigan State University, MI 48824, USA.; 2 Department of Electrical and Computer Engineering, Michigan State University, MI 48824, USA.; 3 Department of Biochemistry and Molecular Biology, Michigan State University, MI 48824, USA.

**Keywords:** COVID-19, SARS-CoV-2, Omicron, infectivity, antibody-resistance, vaccine breakthrough

## Abstract

The Omicron variant of severe acute respiratory syndrome coronavirus 2 (SARS-CoV-2) has rapidly replaced the Delta variant as a dominating SARS-CoV-2 variant because of natural selection, which favors the variant with higher infectivity and stronger vaccine breakthrough ability. Omicron has three lineages or subvariants, BA.1 (B.1.1.529.1), BA.2 (B.1.1.529.2), and BA.3 (B.1.1.529.3). Among them, BA.1 is the currently prevailing subvariant. BA.2 shares 32 mutations with BA.1 but has 28 distinct ones. BA.3 shares most of its mutations with BA.1 and BA.2 except for one. BA.2 is found to be able to alarmingly reinfect patients originally infected by Omicron BA.1. An important question is whether BA.2 or BA.3 will become a new dominating “variant of concern”. Currently, no experimental data has been reported about BA.2 and BA.3. We construct a novel algebraic topology-based deep learning model trained with tens of thousands of mutational and deep mutational data to systematically evaluate BA.2’s and BA.3’s infectivity, vaccine breakthrough capability, and antibody resistance. Our comparative analysis of all main variants namely, Alpha, Beta, Gamma, Delta, Lambda, Mu, BA.1, BA.2, and BA.3, unveils that BA.2 is about 1.5 and 4.2 times as contagious as BA.1 and Delta, respectively. It is also 30% and 17-fold more capable than BA.1 and Delta, respectively, to escape current vaccines. Therefore, we project that Omicron BA.2 is on its path to becoming the next dominating variant. We forecast that like Omicron BA.1, BA.2 will also seriously compromise most existing mAbs, except for sotrovimab developed by GlaxoSmithKline.

## 1 Introduction

On November 26, 2021, the World Health Organization (WHO) declared the Omicron variant (B.1.1.529) of severe acute respiratory syndrome coronavirus 2 (SARS-CoV-2) initially discovered in South Africa a variant of concern (VOC). Within a few days (i.e., December 1, 2021), an artificial intelligence (AI) model predicted the Omicron variant to be about 2.8 times as infectious as the Delta variant, have a near 90% likelihood to escape current vaccines, and severely compromise the efficacy of monoclonal antibodies (mAbs) developed by Eli Lilly, Regeneron, AstraZeneca, and many others, except for GlaxoSmithKline’s sotrovimab [1]. The subsequent experiments confirm Omicron’s high infectivity [2, 3], high vaccine breakthrough rate [4, 5], and severe antibody escape rate [6–8]. The U.S. Food and Drug Administration (FDA) halted the use of mAbs from Eli Lilly and Regeneron in January 2022. Due to its combined effects of high infectivity and high vaccine breakthrough rate, the Omicron variant is far more transmissible than the Delta variant and has rapidly become the dominating variant in the world.

Omicron has three lineages, BA.1 (B.1.1.529.1), BA.2 (B.1.1.529.2), and BA.3 (B.1.1.529.3), which were first detected in November 2021 in South Africa [9]. Among them, BA.1 lineage is the preponderance that has ousted Delta. Compared to the reference genome reported in Wuhan, Omicron BA.1 has a total of 60 mutations on non-structure protein (NSP3), NSP4, NSP5, NSP6, NSP12, NSP14, S protein, envelope protein, membrane protein, and nucleocapsid protein. Among them, 32 mutations are on the spike (S) protein, the main antigenic target of antibodies generated by either infection or vaccination. Fifteen of these mutations affect the receptor-binding domain (RBD), whose binding with host angiotensin-converting enzyme 2 (ACE2) facilitates the viral cell entry during the initial infection [10]. BA.2 shares 32 mutations with BA.1 but has 28 distinct ones. On the RBD, BA.2 has four unique mutations and 12 shared with BA.1. In contrast, the Delta variant has only two RBD mutations. BA.3 shares most of its mutations with BA.1 and BA.2, except for one on NSP6 (A88V). It also has 15 RBD mutations, but none is distinct from BA.1 and BA.2. Nationwide Danish data in late December 2021 and early January 2022 indicate that Omicron BA.2 is inherently substantially more transmissible than BA.1 and capable of vaccine breakthrough [11]. Israel reported a handful of cases of patients who were infected with original Omicron BA.1 strain and have reinfected with BA.2 in a short period [12]. Although BA.2 did not cause worse illness than the original Omicron BA.1 strain, its reinfection is very alarming. It means the antibodies generated from the early Omicron BA.1 were evaded by the BA.2 strain. It is imperative to know whether BA.2 will become the next dominating strain to reinfect the world population.

Studies show that binding free energy (BFE) between the S RBD and the ACE2 is proportional to the viral infectivity [10, 14, 15]. In July 2020, nature selection favoring more infectious variants was discovered as the fundamental law of biology that governs SARS-CoV-2 transmission and evolution [16], including the occurrence of Alpha, Beta, Gamma, Delta, and Omicron variants. Natural selection in SARS-CoV-2 mutations was conformed beyond doubt in April 2021 [17]. Two vital RBD mutation sites, N501 and L452, that later appeared in all main variants, Alpha, Beta, Delta, Gamma, Delta, Epsilon, Theta, Kappa, Lambada, Mu, and Omicron, were also predicted in July 2020 [16]. These discovery and predictions may not be achieved by experimental means.

Currently, there are no experimental results about the infectivity, vaccine breakthrough, and antibody resistance of BA.2 and BA.3 [13]. In this work, we present a comprehensive analysis of Omicron BA.2 and BA.3’s potential of becoming the next prevailing SARS-CoV-2 variant. Our study focuses on the S protein RBD, which is essential for virus cell entry. The RBD is not only crucial for viral infectivity but also essential for vaccines and antibody protections. An antibody that can disrupt the RBD-ACE2 binding would directly neutralize the virus [18–20]. We integrate tens of thousands of mutational and deep mutational data, biophysics, and algebraic topology to construct an AI model. We systematically investigate the binding free energy (BFE) changes of an RBD-ACE2 complex structure and a library of 185 structures of RBD-antibody complexes induced by the RBD mutations of Alpha, Beta, Gamma, Delta, Lambda, Mu, BA.1, BA.2, and BA.3 to reveal their infectivity, vaccine-escape potential, and antibody resistance. Using our comparative analysis, we unveil that the Omicron BA.2 variant is about 1.5 times as infectious as BA.1 and about 4.2 times as contagious as the Delta variant. It also has a 30% higher potential than BA.1 to escape existing vaccines. Therefore, we project the Omicron BA.2 is on its path to becoming the next dominating variant.

## 2 Results

### 2.1 Infectivity

**Figure 1:**
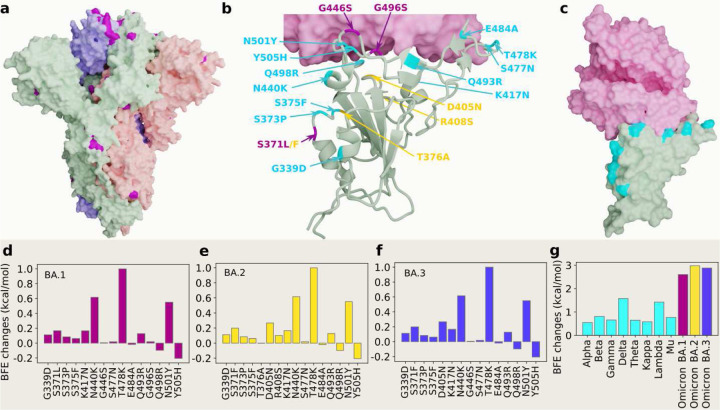
3D structures of Omicron strains, their ACE2 complexes and their mutation-induced BFE changes. **a** Spike protein (PDB: 7WK2 [3]) with Omicron mutations being marked yellow. **b** BA.1 and BA.2 RBD mutations at the RBD-ACE interface (PDB: 7T9L [21]). The shared 12 mutations are labeled in cyan, BA.1 mutations are marked with magenta, and distinct BA.2 mutations are plotted in yellow. **b** The structure of the RBD-ACE2 complex with mutations on cyan spots. **e**, **f** and **g** BFE changes induced by mutations of Omicron BA.1, BA.2, BA.3, respectively. **h** a comparison of predicted mutation-induced BFE changes for few SARS-CoV-2 variants.

Figure 1 a shows the three-dimensional (3D) structure of Omicron BA.1 [3]. At the RBD, Omicron BA.1, BA.2 and BA.3 share 12 RBD mutations, i.e., G339D, S373P, S375F, K417N, N440K, S477N, T478K, E484A, Q493R, Q498R, N501Y, and Y505H as shown in Figure 1 b. However, BA.1 has distinct RBD mutations S371L, G446S, and G496S, BA.2 has S371F, T376A, D405N, and R408S, and BA.3 has S371F, D405N, and G446S. Figures 1 d, e and f present the BFE changes of the RBD-ACE2 complex induced by the RBD mutations of Omicron AB.1, BA.2 and BA.3, respectively. The larger the BFE change is, the higher infectivity will be. Since natural selection favors those mutations that strengthen the viral infectivity [16], the most contagious variant will become dominant in a population under the same competing condition. The accumulated BFE changes are summarized in Figure 1 g. A comparison is given to other main SARS-CoV-2 variants Alpha, Beta, Gamma, Delta, Theta, Kappa, Lambda, and Mu. The Delta variant had the highest BFE change among the earlier variants and was the most infectious variant before the occurrence of the Omicron variant, which explains its dominance in 2021. Omicron BA.1, BA.2, and BA.3 have BFE changes of 2.60, 2.98, and 2.88 kcal/mol, respectively, which are much higher than those of other major SRAS-CoV-2 variants. Among them, Omicron BA.2 is the most infectious variant and is about 20 and 4.2 times as infectious as the original SARS-CoV-2 and the Delta variant, respectively. Our model predicts that BA.2 is about 1.5 as contagious BA.2, which is the same as reported in an initial study [12]. Another report confirms that Omicron BA.2 is more contagious than BA.1 [11]. Therefore, Omicron BA.2 may eventually replace the original Omicron strain BA.1 in the world.

### 2.2 Vaccine breakthrough

Omicron BA.1 is well-known for its ability to escape current vaccines [5, 6]. Its 15 mutations at the RBD enable it to not only strengthen its infectivity by a stronger binding to human ACE2 but also create mismatches for most direct neutralization antibodies generated from vaccination or prior infection. Although BA.1, BA.2, and BA.3 share 12 RBD mutations, BA.1 has 3 additional RBD mutations, BA.2 has 4 additional RBD mutations, and BA.3 has one mutation the same as that of BA.1’s additional ones and two mutations the same as those of BA.2’s additional ones. Therefore, it is important to understand their vaccine-escape potentials. Currently, no experimental result has been reported about the vaccine-breakthrough capability of BA.2 and BA.3.

Experimental analysis of the variant vaccine-escape capability over the world’s populations is subject to many uncertainties. Different vaccines may stimulate different immune responses and antibodies for the same person. Different individuals may have different immune responses and antibodies from the same vaccine due to their different races, gender, age, and underlying medical conditions. Uncontrollable experimental conditions and different experimental methods may also contribute to uncertainties. Consequently, it is impossible to accurately characterize a variant’s vaccine-escape capability (or rate) over the world’s populations.

In our work, we take an integrated approach to understanding the intrinsic vaccine-escape capability of SARS-CoV-2 variants. We collect a library of 185 known antibody and S protein complexes and analyze the mutational impact on the binding of these complexes [1, 22]. The results in terms of mutation-induced BFE changes serve as the statistical ensemble analysis of Omicron subvariants’ vaccine-breakthrough potentials. This molecular-level analysis becomes very useful when it is systematically applied to a series of variants.

**Figure 2:**
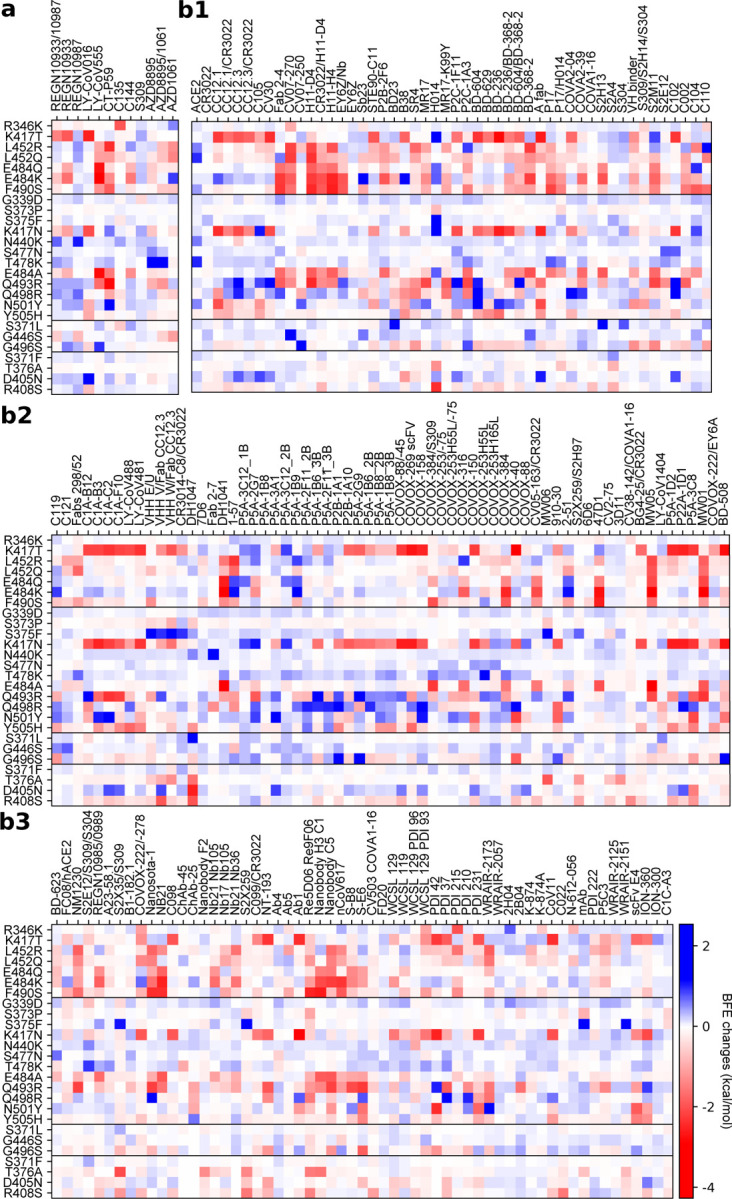
Illustration of mutation-induced BFE changes of 185 antibody-RBD complexes and an ACE2-RBD complex. Positive changes strengthen the binding, while negative changes weaken the binding. **a** Heat map for 12 antibody-RBD complexes in various stages of drug development. Gray color stands for no predictions due to incomplete structures. **b1** Heat map for ACE2-RBD and antibody-RBD complexes. **b2** and **b3** Heat map for antibody-RBD complexes. The first 7 mutations are associated earlier SARS-CoV-2 variants. The next 12 mutations are shared among BA.1, BA.2, and BA.3 strains. The next three mutations are distinct to BA.1, and the final bunch of 4 mutations belong to BA.2.

Figures 2 a, b1, and b2 depict the BFE changes of ACE2-RBD and 185 antibody-RBD complexes induced by the RBD mutations from SARS-CoV-2 variants. The first bunch of 7 mutations is associated with Alpha, Beta, Gamma, Delta, Lambda, and Mu. The second bunch of 12 mutations is shared among BA.1, BA.2, and BA.3. The next bunch of 3 mutations is associated with BA.1. The last bunch of 4 mutations belongs to BA2. Binding-strengthening mutations give rise to positive BFE changes, while binding-weakening mutations lead to negative BFE changes. Obviously, shared Omicron mutations K417N, E484A, and Q493R are very disruptive to many antibodies. BA.1 mutation G496S is also quite disruptive. BA.2 mutations T376A, D405N, and R408S may reduce the efficacy of many antibodies. Apparently, these complexes are significantly impacted by Omicron BA.1, BA.2, and BA.3 RBD mutations. Overall, Figure 2 shows more negative BFE changes than positive ones, suggesting Omicron BA.1, BA.2, and BA3 mutations enable the breakthrough of current vaccines.

**Figure 3:**
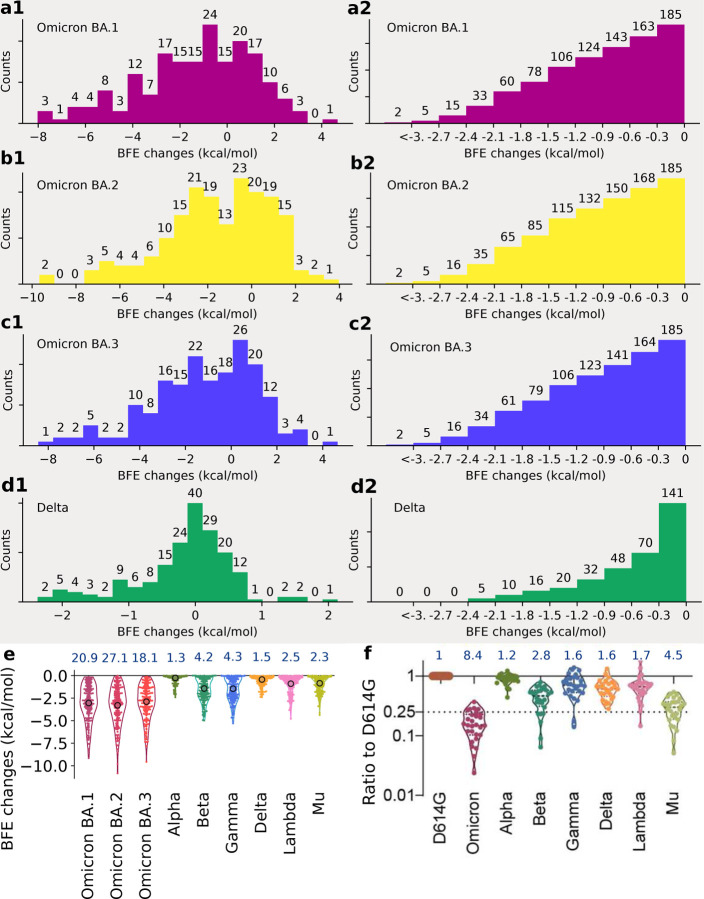
Analysis of variant mutation-induced BFE changes of ACE2-RBD and 185 antibody-RBD complexes. **a1**, **b1**, **c1**, and **d1** The distributions (counts) of accumulated BFE changes induced by Omicron BA.1, BA.2, BA.3, and Delta mutations respectively for 185 antibody-RBD complexes. For each case, there are more mutation-weakened complexes than mutation-strengthened complexes. **a2**, **b2**, **c2**, and **d2** The numbers of antibody-RBD complexes regarded as disrupted by BA.1, BA.2, BA.3, and Delta mutations respectively under different thresholds ranging from 0 kcal/mol, −0.3 kcal/mol, to <−3 kcal/mol. e Accumulated negative BFE changes induced by BA.1, BA.2, BA3, Alpha, Beta, Delta, Gamma, Lambda, and Mu mutations respectively for 185 antibody-RBD complexes. For each variant, the number on the top is the fold of binding affinity reduction computed by *e*^−BFEchange_average_^, where BFEchange_average_, marked by a circle, is the mean value of negative BFE changes for 185 antibody-RBD complexes. **f** The comparison of neutralization activity against Omicron (BA.1), Alpha, Beta, Delta, Gamma, Lambda, and Mu variants based on 28 convalescence sera [5]. For each variant, the number on the top is the ratio of neutralization ED_50_ compared to the reference strain D614G.

Statistical analysis of the BFE changes of 185 antibody-RBD complexes induced by BA.1, BA.2, BA.3, and Delta RBD mutations is presented in Figure 3 and analysis of Alpha, Beta, Gamma, Lambda, and Mu is presented in Figure S2. Accumulated BFE changes are provided in Figure 3 a1, b1, and c1. Obviously, all Omicron subvariants have more negative accumulated BFE changes than positive ones, showing their antibody resistance. Among them, BA.2’s distribution is extended to a wider negative domain, showing its strongest antibody resistance. In contrast, Delta variant’s statistics is given in Figure 3 d1, showing a smaller domain of distribution.

As discussed earlier, it is difficult to obtain a variant’s true vaccine-escape rate over world’s populations. However, a molecular-based comparative analysis can offer desirable information. Figures 3 a2, b2, c2, and d2 depict the number of antibody-RBD complexes that is regarded as disrupted by BA.1, BA.2, BA.3, and Delta mutations, respectively, under different thresholds ranging from 0 kcal/mol, −0.3 kcal/mol, to *<*−3 kcal/mol. Previously, threshold −0.3 kcal/mol was used to decide whether a mutation disrupts an antibody-RBD complex [1], which gives rise to 163, 168, and 164 disrupted antibody-RBD complexes, respectively for BA.1, BA.2, and BA.3. The corresponding rates of potential vaccine breakthrough are 0.88, 0.91, and 0.89 for BA.1, BA.2, and BA.3, respectively. Therefore, BA.2 is slightly more antibody resistant than BA.1. As a reference, the Delta variant may disrupt 70 out of 185 antibody-RBD complexes, suggesting a vaccine-breakthrough rate of 0.37.

It is interesting to compare our analysis with experimental results [5]. In Figure 3 f, the sensitivity of 28 serum samples from COVID-19 convalescent patients infected with an earlier SARS-CoV-2 strain (D614G) was tested against pseudotyped Omicron, Alpha, Beta, Gamma, Delta, Lambda, and Mu [5]. The results indicate the Omicron (BA.1) and Delta variant have 8.4 and 1.6 fold reductions, respectively, to the mean neutralization ED50 of these sera compared with the D614G reference strain. Figure 3 e presents a comparison of accumulated negative BFE changes for variants Omicron BA.1, BA.2, BA.3, Alpha, Beta, Delta, Gamma, Lambda, and Mu. For each antibody-RBD complex, we only consider disruptive effects by setting positive BFE changes to zero and sum over RBD mutations (e.g., 15 mutations for Omicron BA.1 and 2 for Delta) to obtain the accumulated negative BFE change. As such, we have 185 accumulated negative BFE changes for each variant. We use the mean of these 185 values to computed the fold of affinity reduction, which can be compared for different variants against the original virus reported in Wuhan (BFEchange_average_ = 0). The RBD mutations of the Delta variant cause 1.5 fold reduction in the neutralization capability. In the same setting, Omicron BA.1, BA.2, and BA.3 may lead to about 21, 27, and 18 fold increases in their vaccine-breakthrough capabilities. As such, BA.2 is about 30% more capable to escape existing vaccines than BA.1 and 17 times more than the Delta variant. Our prediction has a correlation coefficient of 0.9 with the experiment. With its highest infectivity and highest vaccine-escape potential, the Omicron BA.2 is set to take over the Omicron BA.1 in infecting the world population.

### 2.3 Antibody resistance

The design and discovery of mAbs are part of an important achievement in combating COVID-19. Unfortunately, like vaccines, mAbs are prone to viral mutations, particularly antibody-resistant ones. Early studies predicted that Omicron BA.1 would compromise the anti-COVID-19 mAbs developed by Eli Lilly, Regeneron, AstraZeneca, Celltrion, and Rockefeller University [1]. However, Omicron BA.1’s impact on GlaxoSmithKline’s mAb, called sotrovimab, was predicted to be mild [1]. These predictions have been confirmed and the FDA has halted the use of Eli Lilly and Regeneron’s COVID-19 mAbs. Currently, GlaxoSmithKline’s sotrovimab is the only antibody-drug authorized in the U.S. for the treatment of COVID-19 patients infected by the Omicron variant. An important question is whether sotrovimab remains effective for the BA.2 subvariant that might drive a new wave of infections in the world population.

**Figure 4:**
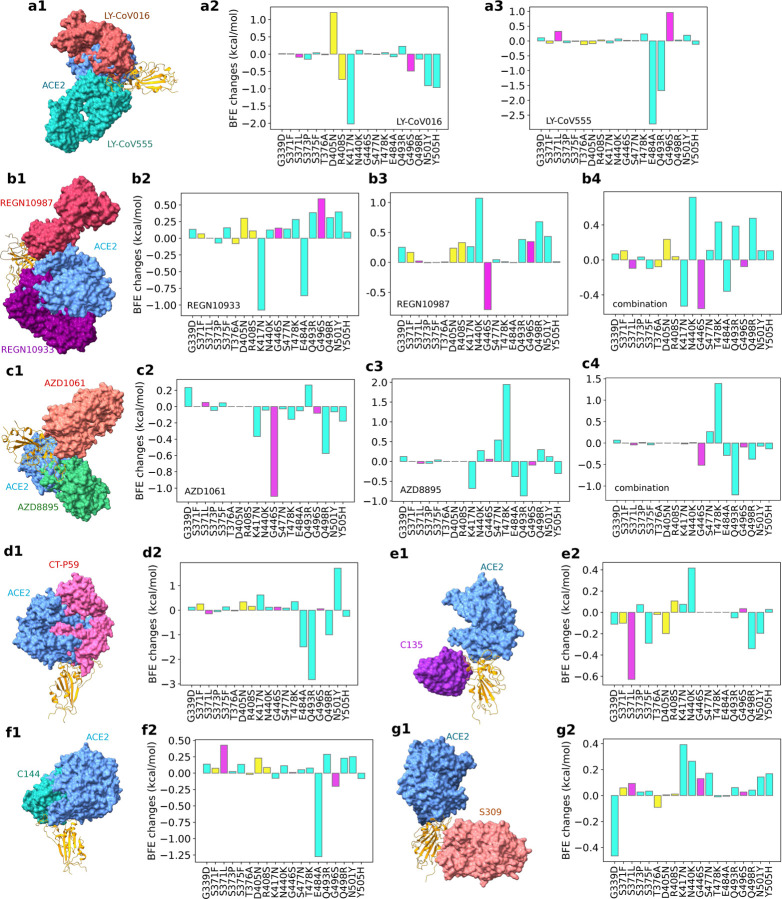
Illustration of Omicron BA.1 and BA.2 RBD mutational impacts on clinical mAbs. **a1**, **b1**, **c1**, **d1**, **e1**, **f1** and **g1** depict the 3D structures of antibody-RBD complexes of Eli Lilly LY-CoV555 (PDB ID: 7KMG [23]) and LY-CoV016 (PDB ID: 7C01 [24]), Regeneron REGN10987 and REGN10933 (PDB ID: 6XDG [25]), AstraZeneca AZD1061 and AZD8895 (PDB ID: 7L7E [26]), Celltrion CT-P59 (aka Regdanvimab, PDB ID: 7CM4), Rockefeller University C135 (PDB ID: 7K8Z) and C144 (PDB ID: 7K90), and GlaxoSmithKline S309 (PDB ID: 6WPS), respectively. In all plots, the ACE2 structure is aligned as a reference. Omicron BA.1 and BA.2 RBD mutation-induced BFE changes (kcal/mol) are given in **a2** and **a3** for Eli Lilly mAbs, b2, b3 and **b4** for Regeneron mAbs, **c2, c3**, and **c4** for AstraZeneca mAbs, **d2** for Celltrion CT-P59, **e2** and **f2** for Rockefeller University mAbs, and **g2** for GlaxoSmithKline S309, respectively. Cyan bars label the BFE changes induced by twelve RBD mutations shared by BA.1, BA.2, and BA.3 subvariants. Magenta bars mark the BFE changes induced by three additional BA.1 RBD mutations. Yellow bars denote the BFE changes induced by four additional BA.2 RBD mutations.

In this work, we further analyze the efficacy of these mAbs for BA.2 and BA.3. Our studies focus on Omicron subvariants’ RBD mutations, which appear to be optimized by the virus to evade host antibody protection and infect the host cell. Figure 4 provides a comprehensive analysis of the BFE changes of various antibody-RBD complexes induced by Omicron BA.1, BA.2, and BA.3. Since BA.3 subvariant’s RBD mutations are the subsets of those of BA.1 and BA.2, we only present 19 unique RBD mutations. Impacts of twelve shared RBD mutations are labeled with cyan, those of three additional BA.1 RBD mutations are marked with magenta, and those of four additional BA.2 RBD mutations are plotted in yellow. Figures 4 a1, b1, c1, d1, e1, f1 and g1 depict 3D antibody-RBD complexes for mAbs from Eli Lilly (LY-CoV016 and LY-CoV555), Regeneron (REGN10933, REGN10987, and REGN10933/10987), AstraZeneca (AZD1061 and AZD8895), Celltrion (CT-P59), Rockefeller University (C135, C144), and GlaxoSmithKline (S309), respectively. The ACE2 is included in these plots as a reference.

Figures 4 a2 and a3 show that LY-CoV016 is disrupted by shared mutation K417N and LY-CoV555 is weakened by shared mutations E484A and Q493R. Additional mutations from BA.2 may not significantly affect Eli Lilly mAbs. However, if BA.2 become dominant, Eli Lilly mAbs would still be ineffective.

The impacts of BA.1 and BA.2 mutations on Regeneron’s mAbs are illustrated in Figures 4 b2, b3 and b4. REGN10933 is undermined by shared mutations N417K and E484A. REGN10987 is disrupted by BA.1 mutation G446S. The antibody cocktail is undermined by shared Omicron mutations as well, which implies Regeneron’s mAbs would still be compromised should Omicron BA.2 become a dominant SRAS-CoV-2 subvariant.

BA.1 and BA.2’s impacts on AstraZeneca’s AZD1061 and AZD8895 are demonstrated in Figures 4 c2, c3 and c4. It is noticed that BA.1 mutation G446S has a disruptive effect on AZD1061. AZD8895 is weakened by two shared mutations. The AZD1061-AZD8895 combination is also disrupted by shared mutation Q493R. Therefore, the efficacy of AstraZeneca’s mAbs would be reduced should BA.2 prevail in world populations.

As shown in Figure 4 d2, Celltrion’s mAb CT-P59 is prone to shared mutations Q493R and E484A. BA.2 mutations may not bring additional destruction. However, the shared mutations pose a threat to Celltrion’s mAb, which implies its efficacy would not restore should BA.2 prevail.

Figures 4 e2 and f2 present BA.1 and BA.2’s mutational impacts on Rockefeller University’s mAbs. C135 is mainly disrupted by Omicron BA.1 and its C144 is made ineffective by shared mutation E484A. Therefore, C135 might become effective if BA.2 dominates.

Finally, we plot mutational impacts on antibody S309’s binding with RBD in Figure 4 g2. Antibody S309 is the parent antibody for Sotrovimab developed by GlaxoSmithKline and Vir Biotechnology, Inc. It is seen from the figure that there is only one disruptive BFE change of −0.47kcal/mol and the rest of the BFE changes are mostly positive. The BA.2 mutations have little effect on S309. Therefore, we expect a mild effect from Omicron BA.1 and BA.2 on sotrovimab.

It is interesting to understand why S309 is the only antibody that is not significantly affected by Omicron variants. Figure 4 show that all mAbs that compete with the human ACE2 for the receptor-binding motif (RBM) are seriously compromised by Omicron subvariants because most of the RBD mutations locate at the RBM. A possible reason is that Omicron subvariants had optimized RBD mutations at the RBM to strengthen the viral infectivity and evade the direct neutralization antibodies. Consequently, all mAbs that target RBM are seriously compromised by Omicron subvariants. Figures 4 e1 and g1 show that antibodies C135 and S309 do not directly compete with ACE2 for the RBM. However, C135 is still very close to the RBM and significantly weakened by some Omicron mutations. In contrast, S309 is further away from the RBM and escapes from Omicron’s RBD mutations.

## 3 Materials and Methods

The deep learning model is designed for predicting mutation-induced BFE changes of the binding between protein-protein interactions. A series of three steps consist of training data preparation, feature generations, and deep neural network training and prediction (see Figure S2). Here, we briefly discuss each step and leave more details in Supporting Information. Readers are also suggested literature [16, 27, 28] for more details.

Firstly, the training data is prepared to comprise experimental BFE changes and next-generation sequencing data. SKEMPI 2.0 [29] is the fundamental BFE change dataset. Additionally, SARS-CoV-2 related datasets are the mutational scanning data of the ACE2-RBD complex [30–32] and the CTC-445.2-RBD complex [32]. Next is to prepare the features. It is required a variety of biochemical, biophysical, and mathematics features from PPI complex structures, such as surface areas, partial charges, van der Waals interaction, Coulomb interactions, pH values, electrostatics, persistent homology, graph theory, etc. [16, 33] A detailed list and description of these features are provided in Supporting Information. In the following, the key idea of the element-specific and site-specific persistent homology is illustrated briefly. As the persistent homology [34, 35] introduced as a useful tool for data analysis for scientific and engineering applications, it is further applied to molecular studies [27, 36]. For 3D structures, atoms are modeled as vertices in a point cloud. Then edges, faces, etc. can be constructed as simplices *σ* which form simplicial complexes *X*. Groups *C*_*k*_(*X*), *k* = 0, 1, 2, 3 are sets of all chains of *k*th dimension, which is defined as a finite sum of simplices as $\sum_{i}\alpha_{i}\sigma_{i}^{k}$ with coefficients *α*_*i*_. The boundary operator *∂*_*k*_ therefore, maps $C_{k}\left(X\right)\to C_{k-1}\left(X\right)$ as

(1) $$\partial_{k}\sigma^{k}=\sum_{i=0}^{k}{\left(-1\right)}^{i}\left[v_{0},\cdots ,{\hat{v}}_{i},\cdots ,v_{k}\right],$$

where $\sigma^{k}=\left\{v_{0},\cdots ,v_{k}\right\}$ and $\left[v_{0},\cdots ,{\hat{v}}_{i},\cdots ,v_{k}\right]$ is a (*k*−1)-simplex excluding *v*_*i*_ with *∂*_*k*−1_*∂*_*k*_ = 0. The chain complex is given as

(2) $$\cdots \overset{\partial_{k+1}}{\to }C_{k}\left(X\right)\overset{\partial_{k}}{\to }C_{k-1}\left(X\right)\overset{\partial_{k-1}}{\to }\cdots \overset{\partial_{2}}{\to }C_{1}\left(X\right)\overset{\partial_{1}}{\to }C_{0}\left(X\right)\overset{\partial_{0}}{\to }0.$$

The *k*-th homology group *H*_*k*_ is defined by *H*_*k*_ = *Z*_*k*_*/B*_*k*_ where *Z*_*k*_ = ker *∂*_*k*_ = {*c* ∈ *C*_*k*_ | *∂*_*k*_*c* = 0} and *B*_*k*_ = im *∂*_*k*+1_ = {*∂*_*k*+1_*c* | *c* ∈ *C*_*k*+1_}. Thus, the Betti numbers can be defined by the ranks of *k*-th homology group *H*_*k*_. Persistent homology can be devised to track Betti numbers through a filtration where *β*_0_ describes the number of connected components, *β*_1_ provides the number of loops, and *β*_2_ is the number of cavities. Therefore, using persistent homology, the atoms of 3D structures are grouped according to their elements, as well as the atoms from the binding site of antibodies and antibodies. The interactions and their impacts on PPI complex bindings are characterized by the topological invariants, which are further implemented for machine learning training.

Lastly, a deep learning algorithm, artificial/deep neural networks (ANNs or DNNs), is used to tackle the features with datasets for training and predictions [28]. A trained model is available at TopNetmAb, a SARS-CoV-2-specific model, whose early model was integrating convolutional neural networks (CNNs) with gradient boosting trees (GBTs) and was trained only on the SKEMPI 2.0 dataset with a high accuracy [33].

Recent work with predictions from TopNetmAb [22, 28, 37] is highly consistent with experimental results. One should notice it is important with the help of the aforementioned deep mutational datasets related to SARS-CoV-2. The Pearson correlation of our predictions for the binding of CTC-445.2 and RBD with experimental data is 0.7 [28, 32]. Meanwhile, a Pearson correlation of 0.8 is observed of the predictions of clinical trial antibodies against SARS-CoV-2 induced by emerging mutations in the same work [28] compared to the natural log of experimental escape fractions [38]. Moreover, the prediction of single mutations L452R and N501Y for the ACE2-RBD complex have a perfect consistency with experimental luciferase data [28,39]. More detailed validations are in Supporting Information.

## 4 Conclusion

The Omicron variant has three subvariants BA.1, BA.2, and BA3. The Omicron BA.1 has surprised the scientific community by its large number of mutations, particularly those on the spike (S) protein receptor-binding domain (RBD), which enable its unusual infectivity and high ability to evade antibody protections induced by viral infection and vaccination. Viral RBD interacts with host angiotensin-converting enzyme 2 (ACE2) to initiate cell entry and infection and is a major target for vaccines and monoclonal antibodies (mAbs). Omicron BA.1 exploits its 15 RBD mutations to strengthen its infectivity and disrupt mAbs generated by prior viral infection or vaccination. Omicron BA.2 and BA.3 share 12 RBD mutations with BA.1 but differ by 4 and 3 RBD mutations, respectively, suggesting potentially serious threats to human health. However, no experimental result has been reported for Omicron BA.2 and BA.3, although BA.2 is found to be able to alarmingly reinfect patients originally infected by Omicron BA.1 [12]. In this work, we present deep learning predictions of BA.2’s and BA.3’s potential to become another dominating variant. Based on an intensively tested deep learning model trained with tens of thousands of experimental data, we investigate Omicron BA.2’s and BA.3’s RBD mutational impacts on the RBD-ACE2 binding complex to understand their infectivity and a library of 185 antibodies to shed light on their threats to vaccines and existing mAbs. We unveil that BA.2 is about 1.5 and 4.2 times as contagious as BA.1 and Delta, respectively. It is also 30% and 17-fold more capable than BA.1 and Delta, respectively, to escape current vaccines. It is predicted to undermine most existing mAbs, except for sotrovimab developed by GlaxoSmithKline. We forecast Omicron BA.2 will become another prevailing variant by infecting populations with or without antibody protection.

## Supplementary Material

Supplement 1
